# Concordance of cribriform architecture in matched prostate cancer biopsy and radical prostatectomy specimens

**DOI:** 10.1111/his.13893

**Published:** 2019-08-02

**Authors:** Eva Hollemans, Esther I Verhoef, Chris H Bangma, Ivo Schoots, John Rietbergen, Jozien Helleman, Monique J Roobol, Geert J L H van Leenders

**Affiliations:** ^1^ Department of Pathology Erasmus MC University Medical Center Rotterdam the Netherlands; ^2^ Department of Urology Erasmus MC University Medical Center Rotterdam the Netherlands; ^3^ Department of Radiology Erasmus MC University Medical Center Rotterdam the Netherlands; ^4^ Department of Urology Franciscus Gasthuis and Vlietland Rotterdam the Netherlands

**Keywords:** biopsy, cribriform, outcome, prostate cancer, radical prostatectomy

## Abstract

**Aims:**

Invasive cribriform and/or intraductal carcinoma have been identified as independent adverse parameters for prostate cancer outcome. Little is known on biopsy undersampling of cribriform architecture. Our aim was to determine the extent of cribriform architecture undersampling and to find predictive factors for identifying false cribriform‐negative cases.

**Methods and results:**

We reviewed 186 matched prostate biopsies and radical prostatectomy specimens. Of 97 biopsy grade group 2 (Gleason score 3 + 4 = 7) patients, 22 (23%) had true cribriform‐negative (TN), 39 (40%) false‐negative (FN) and 36 (37%) true‐positive (TP) biopsies. Patients with FN biopsies had higher, although not statistically significant (*P* = 0.06), median PSA levels than patients with TP biopsies (12 versus 8 ng/ml). A PI‐RADS 5 lesion was present in nine of 16 (54%) FN and three of 11 (27%) TN biopsies (*P* = 0.05). Positive biopsy rate (*P* = 0.47), percentage Gleason pattern 4 (*P* = 0.55) and glomeruloid architecture (*P* = 1.0) were not different. Logistic regression identified PSA as an independent predictor (odds ratio = 3.5; 95% confidence interval = 1.2–9.4, *P* = 0.02) for cribriform architecture on radical prostatectomy, but not PI‐RADS score. The FN rate for large cribriform architecture at radical prostatectomy was 27%, which was lower than for any cribriform architecture (*P* = 0.01). During follow‐up (median 27 months), biochemical recurrence‐free survival of patients with TP biopsies was significantly shorter than that of those with FN biopsies (*P* = 0.03).

**Conclusion:**

In conclusion, 40% of grade group 2 prostate cancer biopsies were FN for cribriform architecture. These patients had higher PSA levels and more frequent PI‐RADS score 5 lesions than men with TN biopsies.

## Introduction

Risk stratification and therapeutic decision‐making in prostate cancer patients is affected by potential biopsy undersampling. The Gleason score is one of the most important parameters for predicting disease outcome and guiding individual treatment. Men with Gleason score 3 + 3 = 6 (International Society of Urological Pathology (ISUP) grade group 1] prostate cancer are eligible for active surveillance, whereas men with Gleason score ≥4 + 3 = 7 (grade groups 3–5) are usually treated with radical prostatectomy, radiation therapy and/or hormonal therapy. The optimal therapeutic strategy for men with Gleason score 3 + 4 = 7 (grade group 2) is still a matter of debate. While most of these patients will undergo active treatment, surveillance is increasingly being considered in this subgroup. Incorporation of additional clinicopathological and molecular parameters might be able to support optimal decision‐making in this large prostate cancer subpopulation.

Grade group 2 prostate cancer is a heterogeneous disease with variable architectural growth patterns and Gleason pattern 4 quantities. While individual growth patterns are not routinely mentioned in pathology reports, recent studies have shown that patients with cribriform architecture have an adverse outcome compared to those without.^1^, ^2^, ^3^ Both invasive and intraductal cribriform architecture have been associated with adverse clinicopathological characteristics, post‐operative recurrence rates, metastasis and disease‐specific death.^4^, ^5^, ^6^, ^7^, ^8^ Conversely, biopsy grade group 2 prostate cancer patients without cribriform architecture have comparable disease‐specific survival and post‐operative biochemical recurrence rates to men with grade group 1 disease.^1^, ^19^ Quantification of Gleason pattern 4 can further add in risk stratification, as post‐operative biochemical recurrence rates increment with a higher Gleason pattern 4 tumour percentage.^10^ Cribriform architecture and Gleason pattern 4 quantification might therefore be important adjuncts in risk stratification of grade group 2 prostate cancer patients.

While pathological tumour characteristics are important for clinical decision‐making, prostate biopsies are prone to undersampling. Prostate cancer is up‐graded in up to 40% of subsequent radical prostatectomy specimens.^11^, ^12^ At present, little is known on the extent of undersampling in detection of cribriform architecture or Gleason pattern 4 percentage. The aim of our study is to determine the extent of undersampling for the detection of cribriform architecture in matched prostate biopsy and radical prostatectomy specimens, and to identify potential factors for discriminating true‐ from false cribriform‐negative prostate biopsies.

## Materials and methods

### Patient selection

We identified 186 patients who had undergone both biopsy and subsequent radical prostatectomy at Erasmus MC University Medical Center, Rotterdam, the Netherlands between 2010 and 2017. Biopsies were prompted by elevated prostate‐specific antigen (PSA) levels or obtained in the scope of active surveillance. The Prostate Imaging Reporting and Data System (PI‐RADS) score was annotated by an expert uroradiologist, when patients had received multiparametric magnetic resonance imaging (MRI).^13^ When suspicious lesions (PI‐RADS 3–5) were visible on MRI, targeted MRI‐ultrasound fusion biopsies were taken. Individual biopsy cores were enclosed in separate containers and radical prostatectomy specimens were completely embedded for diagnostic purposes. All slides of both biopsies and radical prostatectomies were available for pathological review. This study was approved by the institutional Medical Research Ethics Committee (MEC‐2018‐1614).

### Pathological evaluation

All biopsies were reviewed by three investigators, who were blinded to clinical outcome and radical prostatectomy characteristics. For each biopsy core the following features were recorded: Gleason score, grade groups according to the World Health Organisation (WHO)/ISUP 2014 guidelines, maximal single biopsy tumour length (mm), overall estimated percentage Gleason pattern 4 and individual tumour growth patterns.^14^ Invasive cribriform Gleason pattern 4 was not distinguished from intraductal carcinoma because of their significant morphological overlap, which would require extensive immunohistochemical staining for further discrimination.^1^ If targeted biopsies were obtained, these were considered as separate biopsies and not as one single biopsy. Matching radical prostatectomy specimens were evaluated as described previously.^4^ We recorded Gleason score, grade group, pT‐stage according to the AJCC TNM 8th edition, surgical margin status, percentage Gleason pattern 4 and individual growth patterns.^15^ Furthermore, we distinguished small and large expansive cribriform growth pattern based on a cut‐off of two times the size of adjacent pre‐existent normal glands.^4^

### Clinical follow‐up

After radical prostatectomy, clinical follow‐up consisted of bi‐annual, and later annual monitoring of serum PSA levels. Biochemical recurrence was defined as PSA levels ≥0.2 ng/ml measured at two consecutive time‐points at least 3 months apart with undetectable PSA levels after operation, or as PSA increase of >2.0 ng/ml when serum PSA had not declined to zero after operation. Survival was defined as time in months from radical prostatectomy to biochemical recurrence or last follow‐up.

### Statistical analysis

Continuous variables with normal distribution were compared by Student’s *t*‐test and one‐way analysis of variance (anova) and those without normal distribution with the Mann–Whitney *U*‐test. For categorical parameters, ^2^ or Fisher’s exact were used. Correlation between continuous variables was analysed using Pearson’s correlation coefficient. Dichotomous outcome variables were analysed using logistic regression. Survival was visualised by Kaplan–Meier curves. Statistics were performed using R version 3.2.2 (R Foundation for Statistical Computing, Vienna, Austria) and results were considered significant when the two‐sided *P*‐value was <0.05.

## Results

### Clinicopathological characteristics

The entire cohort consisted of 186 patients with matched biopsy and radical prostatectomy specimens. The mean age at time of operation was 65 years [interquartile range (IQR) = 62–70] and the mean PSA level was 12 ng/ml (IQR = 6–15). In total, 144 (77%) patients underwent systematic biopsies, 26 (14%) received systematic and targeted biopsies and 16 (9%) had targeted biopsies only. The mean number of biopsies taken was nine (IQR = 8–10), with four (IQR = 3–5) biopsies containing adenocarcinoma, representing 49% (IQR = 30–66) of the total number of biopsy cores. Fifty (27%) patients had overall biopsy grade group 1, 99 (53%) grade group 2, 11 (6%) grade group 3, 15 (8%) grade group 4 and 11 (6%) grade group 5.

On radical prostatectomy, 87 (47%) adenocarcinomas were pT2, 76 (41%) pT3a and 23 (12%) pT3b. Distribution of the grade groups on radical prostatectomy was as follows: 19 (10%) grade group 1, 108 (58%) grade group 2, 25 (14%) grade group 3, 17 (9%) grade group 4 and 17 (9%) grade group 5. Tumour up‐grading occurred in 65 (35%) and down‐grading in 14 (8%) radical prostatectomies, while 107 (57%) cases had concordant tumour grades. Positive surgical margins were present in 63 (34%) patients. Eighty patients had simultaneously undergone pelvic lymph node dissection, 18 of which (23%) contained lymph node metastasis. The mean post‐operative follow‐up was 32 months (median = 22, IQR = 8–51).

Invasive cribriform and/or intraductal carcinoma was observed in 57 (31%) diagnostic biopsies and in 128 (69%) radical prostatectomy specimens (Table 1). Cribriform architecture was present in both matched biopsy and radical prostatectomy specimens in 55 (30%) and absent in 56 (30%) cases. In 73 (39%) men cribriform architecture was observed in the radical prostatectomy specimen, but not in preceding biopsies. Two cases (1%) had cribriform architecture at biopsy but not at subsequent radical prostatectomy, which is probably due to sampling error, and these were excluded from further analyses. Therefore, the sensitivity for cribriform architecture on biopsies was 43%, while specificity was 97%. Cribriform architecture was observed more frequently in targeted (19 of 40; 48%) than systematic biopsies (36 of 144; 25%, *P* = 0.01).

**Table 1:** Prevalence of invasive cribriform and/or intraductal carcinoma (CR/IDC) in biopsies and matched radical prostatectomies

	Radical prostatectomy	
Prostate biopsy	CR/IDC−	CR/IDC+
CR/IDC−	56 (30%)	73 (39%)
CR/IDC+	2 (1%)	55 (30%)

### Concordance of cribriform architecture in grade group 2 prostate cancer biopsies

Because cribriform architecture might be most relevant for treatment decisions in patients with biopsy grade group 2 prostate cancer, we performed further analyses within this subgroup (*n* = 97). Thirty‐six (37%) patients with biopsy grade group 2 demonstrated cribriform architecture on both matched biopsy and radical prostatectomy specimen (true cribriform‐positive, CR+/CR+), while cribriform architecture was absent in both specimens in 22 (23%) cases (true cribriform‐negative, CR−/CR−). In 39 (40%) patients, cribriform architecture was present on radical prostatectomy but not on preceding biopsy; these patients were considered as having false cribriform‐negative (CR−/CR+) biopsies. None of the patients with biopsy grade group 2 had cribriform architecture on biopsy, while radical prostatectomy was negative for cribriform architecture.

### Identification of predictors in true‐ and false cribriform‐negative grade group 2 prostate cancer biopsies

Patients with true‐negative biopsies were slightly younger (62 versus 65 years, *P* = 0.06) and had lower PSA levels (8 versus 12 ng/ml, *P* = 0.06) than men with false‐negative biopsies; however, these differences were not significant (Table 2). In total, 51 patients (53%) had undergone multiparametric MRI prior to biopsy. Of 11 patients with true‐negative biopsies, three (27%) had a PI‐RADS 5 lesion compared to nine of 16 (56%) false‐negative and 17 of 24 (71%) true‐positive biopsy patients (*P* = 0.05). The number of biopsies (*P* = 0.53), percentage of positive biopsies (*P* = 0.47) and maximal tumour length (*P* = 0.44) were not different between true‐ and false‐negative biopsies. As Gleason pattern 4 percentage and glomeruloid architecture have both been associated with cribriform architecture, we assessed the predictive value of these pathological parameters.^8^, ^16^ Mean percentage of Gleason pattern 4 was 12% (IQR = 5–10%) in true‐negative biopsies and 11% (IQR = 5–16%) in false‐negative biopsies (*P* = 0.55). There was only a weak correlation between percentage Gleason pattern 4 on biopsies (mean = 13%, IQR = 5–20%) and matched radical prostatectomies (mean = 31%, IQR = 10–40%, *R* ^2^ = 0.093; *P* = 0.001). Glomeruloid growth pattern was encountered in six of 22 (27%) true‐negative and 11 of 39 (28%) false‐negative biopsies (*P* = 1.0).

**Table 2:** Characteristics of biopsy grade group 2 prostate cancer (PCa) patients stratified for true cribriform‐negative (CR−/CR−), false cribriform‐negative (CR−/CR+) and true cribriform‐positive (CR+/CR+) biopsies

	CR−/CR− (*n* = 22)	CR−/CR+ (*n* = 39)	CR+/CR+ (*n* = 36)	*P*‐value
Age (years)	62 (63, 58–65)	65 (66, 62–71)	66 (66, 62–71)	0.06^a^
PSA	8 (8, 6–10)	12 (10, 6–17)	16 (13, 9–19)	0.06^b^
PI‐RADS score				
No MRI	11 (50%)	23 (59%)	12 (33%)	0.10^c^
1–2	3 (14%)	0 (0%)	0 (0%)	
3	1 (5%)	1 (3%)	2 (6%)	
4	4 (18%)	6 (15%)	5 (14%)	
5	3 (14%)	9 (23%)	17 (47%)	
Number of biopsies	9 (9, 8–10)	8 (8, 7–10)	10 (10, 8–12)	0.53^d^
Number PCa‐positive biopsies	4 (3, 2–6)	4 (4, 3–5)	6 (5, 4–8)	0.64^d^
Percentage PCa‐positive biopsies	47 (38, 25–71)	52 (50, 31–73)	59 (61, 40–76)	0.47^d^
Maximal tumour length (mm)	7 (7, 5–8)	8 (7, 5–10)	9 (10, 7–12)	0.44^d^
Percentage Gleason pattern 4	12 (8, 5–10)	11 (8, 5–16)	17 (15, 7–23)	0.55^a^
Presence of glomeruloid growth	6 (27%)	11 (28%)	12 (33%)	1.0^e^
Presence of large cribriform growth	0	6 (15%)	16 (44%)	NA
Presence of targeted biopsies	2 (9%)	8 (20%)	13 (36%)	0.30^e^
ISUP grade on radical prostatectomy				
1	2 (9%)	1 (3%)	1 (3%)	0.01^e^
2	18 (82%)	29 (74%)	26 (72%)	
3	0 (0%)	8 (20%)	7 (19%)	
4	0 (0%)	1 (3%)	1 (3%)	
5	2 (9%)	0 (0%)	1 (3%)	
Positive surgical margins	8 (36%)	12 (31%)	12 (33%)	0.78^c^
pT stage (TNM 8th)				
2	11 (50%)	15 (38%)	17 (47%)	0.66^c^
3a	10 (45%)	20 (51%)	12 (33%)	
3b	1 (5%)	4 (11%)	7 (20%)	
Biochemical recurrence	2 (9%)	6 (15%)	13 (36%)	0.69^e^
Metastasis	0 (0%)	1 (3%)	4 (11%)	NA

Mean (median, interquartile range) or *n* (%).

NA, not applicable; PSA, prostate‐specific antigen; MRI, magnetic resonance imaging; PI‐RADS, Prostate Imaging Reporting and Data System.

a Wilcox‐test

b *t*‐test (log2 values were used for this test)

c χ^2^.

d one‐way analysis of variance (anova)

e Fisher’s test. *P*‐values resemble comparison between CR−/CR− and CR−/CR+.

Logistic regression analysis on cribriform‐negative biopsy patients showed that age [odds ratio (OR) = 1.1, 95% confidence interval (CI) = 1.0–1.3, *P* = 0.02] and PSA (OR = 3.3, 95% CI = 1.2–9.1, *P* = 0.02) were independent predictive parameters for presence of cribriform architecture on radical prostatectomy in multivariable analysis, whereas PI‐RADS score, number and percentage of positive biopsies, maximal tumour length, presence of targeted biopsies and percentage Gleason grade 4 were not (Table 3).

**Table 3:** Logistic regression analysis of biopsy grade group 2 cribriform‐negative prostate cancer (PCa) patients (*n* = 61), predicting cribriform architecture on radical prostatectomy

	Univariate			Multivariable		
	OR	95% CI	*P*‐value	OR	95% CI	*P*‐value
Age (years)	1.1	1.0–1.2	0.06	1.1	1.0–1.3	0.02
PSA (log2)	2.2^a^	1.0–4.8	0.04	3.3^a^	1.2–9.1	0.02
PI‐RADS score						
<5	Ref.					
5	1.9	0.5–7.9	0.38	1.8	0.3–9.1	0.49
Number of biopsies	0.9	0.8–1.1	0.53	0.8	0.6–1.1	0.21
Percentage of PCa‐positive biopsies	2.1	0.3–15	0.47	0.2	0.0–5.5	0.35
Maximal tumour length (mm)	1.1	0.9–1.2	0.43	1.0	0.9–1.3	0.70
Percentage of Gleason pattern 4	1.0	0.9–1.0	0.70	1.0	0.9–1.0	0.36
Presence of targeted biopsies						
No	Ref.					
Yes	2.6	0.5–13	0.26	1.1	0.1–10	0.91

OR, odds ratio; CI, confidence interval; PI‐RADS, Prostate Imaging Reporting and Data System.

a Per doubling unit.

### Comparison of false‐negative and true cribriform‐positive grade group 2 biopsies

PSA levels of men with true‐positive biopsies were slightly higher than of those with false‐negative biopsies, but this was not statistically significant (16 versus 12 ng/ml, *P* = 0.13). Patients with true‐positive biopsies had a significantly higher total number of biopsies (10 versus 8, *P* = 0.02) and number of tumour‐positive biopsies (6 versus 4, *P* = 0.001); however, no differences were seen in percentage of positive biopsies (59 versus 52%, *P* = 0.19) when compared to patients with false‐negative biopsies. The percentage of Gleason pattern 4 was higher in patients with cribriform‐positive biopsies than in those with false‐negative biopsies (17 versus 11%, *P* = 0.03). Final grade group (*P* = 0.97), pT stage (*P* = 0.27) and surgical margin status (*P* = 0.24) of the radical prostatectomy specimens were not different between these two groups. The tumour volume percentage of cribriform growth at radical prostatectomy was higher in patients with true‐positive biopsies than in those with false‐negative biopsies, but this did not meet conventional measures of significance (13% versus 6%, *P* = 0.06).

Large expansile cribriform architecture, which represents an aggressive subtype of invasive cribriform carcinoma, was present in 22 of 97 (23%) radical prostatectomy specimens.^4^ Sixteen of these 22 (73%) patients had any size cribriform fields on biopsy, while biopsies were false‐negative in six (27%) men. The false‐negative rate for more aggressive large cribriform architecture (six of 22; 27%) was lower than for any cribriform architecture (39 of 75; 52%, *P* = 0.01). If large cribriform carcinoma was present at radical prostatectomy, the tumour volume percentage of any cribriform growth in the operation specimens did not differ between men with false cribriform‐negative and true‐positive biopsies (*P *= 0.5). This indicates that the lower false‐negative rate of large cribriform growth was not merely due to a larger total cribriform tumour percentage at radical prostatectomy.

### Clinicopathological outcome in grade group 2 patients

Of 97 patients with biopsy grade group 2 prostate cancer, 73 (75%) had concordant grade group at radical prostatectomy, 20 (21%) were up‐graded to grade groups 3 to 5 and four (4%) down‐graded to grade group 1. Up‐grading occurred in nine of 36 (25%) true‐positive and in nine of 39 (23%) false‐negative biopsies, and was significantly lower (*P* = 0.01) in true‐negative biopsies (two of 22, 9%). Extraprostatic expansion and surgical margins status were not significantly different between the three groups.

Biochemical recurrence occurred in 21 (22%) patients and was significantly more frequent in the true‐positive (13 of 36, 36%) than in the false‐negative group (six of 39, 15%, *P* = 0.03). The true‐negative group (two of 22, 9%) showed the lowest incidence of biochemical recurrence; however, this difference was not significant (*P* = 0.13) when compared to the false‐negative group.

**Figure 1:**
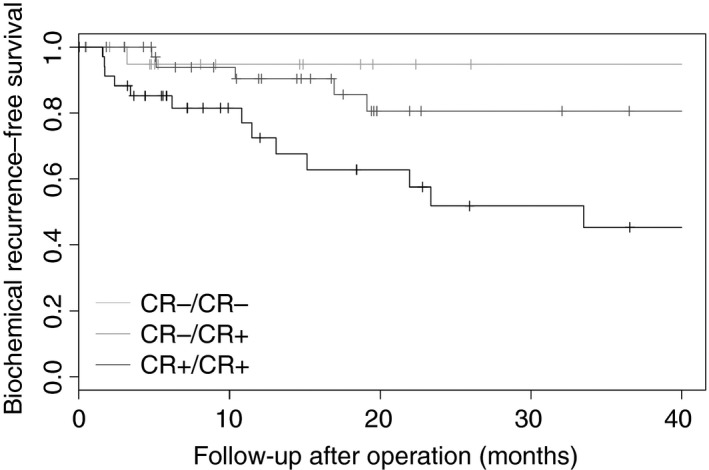
Biochemical recurrence‐free survival of biopsy grade group 2 prostate cancer patients, stratified for the presence of cribriform architecture on biopsies and subsequent radical prostatectomies (log‐rank over all groups, *P*‐value = 0.02).

The median post‐operative follow‐up of grade group 2 patients was 27 months (mean = 18, IQR = 6–40). Patients experienced biochemical recurrence after a median of 14 months (mean = 24, IQR = 5–32). Biochemical recurrence‐free survival was not significantly different between patients with true‐negative and false‐negative biopsies (log‐rank *P* = 0.55). Patients with cribriform‐positive biopsies had significantly shorter biochemical recurrence‐free survival than men with false‐negative biopsies (log‐rank *P* = 0.03, Figure 1).

## Discussion

Identification and pathological reporting of invasive cribriform and/or intraductal carcinoma of the prostate are increasingly important, as they are both associated with adverse clinical outcome.^1^, ^2^, ^5^, ^6^ Biopsy undersampling is a well‐known problem which might have a significant impact on individual patient management.^11^, ^17^, ^18^ Hitherto, little is known about biopsy undersampling in identifying cribriform architecture. In this study we demonstrated that biopsies were false‐negative for cribriform architecture in 39% of all cases and in 40% of patients with biopsy grade group 2 prostate cancer. In false‐negative grade group 2 patients, age and PSA level were independent predictive parameters for the presence of cribriform architecture on subsequent radical prostatectomy, while the percentage of positive biopsies, maximal biopsy tumour length, percentage Gleason pattern 4 and glomeruloid growth were not. Patients with the more aggressive large cribriform growth pattern on radical prostatectomy were, however, less likely to have cribriform‐negative biopsies.^4^ Biopsy grade group 2 patients with false cribriform‐negative biopsies showed better biochemical recurrence‐free survival rates than men with true cribriform‐positive biopsies, although follow‐up was relatively short.

Masoomian *et al*.^19^ studied concordance rates of cribriform architecture in 245 matched biopsies and operation specimens and found a relatively low sensitivity of 47%, corresponding well with the 43% sensitivity in our study. In their subset of grade group 2 biopsy patients, false‐negative and true‐positive biopsies both had a more advanced stage compared to true‐negative biopsies on radical prostatectomy, suggesting that men with false‐negative and true‐positive biopsies have a comparable outcome. This contrasts with our study, as we found that post‐operative biochemical recurrence‐free survival of men with true‐positive biopsies was significantly shorter than of those with false‐negative biopsies. The difference might be explained by the different and relatively small cohorts in both studies.

While most patients with biopsy grade group 2 prostate cancer undergo active treatment, the question arises as to whether surveillance could be a safe alternative for subgroups of this large patient population. It has, for instance, been proposed that patients with biopsy grade group 2 prostate cancer and low Gleason pattern 4 percentage should be considered for surveillance.^20^, ^21^ Others have suggested that biopsy grade group 2 prostate cancer patients without invasive cribriform and/or intraductal carcinoma might be eligible for surveillance.^7^, ^9^ To further support clinical decision tools, it is important to gain insight into the false‐negative rate of potentially aggressive disease parameters and to determine how this rate can be minimised to an acceptable level. In the current study, we showed that consideration of PSA level, which is an important parameter for active surveillance, might prevent men with potentially aggressive false‐negative biopsies from being abstained from immediate treatment. Furthermore, presence of a PI‐RADS 5 lesion on multiparametric MRI might also be indicative of more aggressive disease. Truong *et al*.^22^ identified cribriform morphology in combined systematic and targeted biopsies in 37% of PI‐RADS 5, 24% of PI‐RADS 4 and 6% of PI‐RADS 2 lesions, suggesting that high‐grade MRI lesions are related to more aggressive tumours with cribriform morphology. Prendeville *et al*.^23^ identified cribriform morphology in 8% of PI‐RADS 3/4 lesions and in 39% of PI‐RADS 5 lesions, indicating that PI‐RADS score might be a predictor for cribriform‐positive prostate cancer. In this study we showed that 56% of false‐negative biopsies had a PI‐RADS 5 lesion compared to 27% of true‐negative biopsies. However, due to the small number of patients who had undergone MRI, PI‐RADS score was not a predictor for cribriform architecture in logistic regression analysis.

We were not able to find any predictive value of biopsy percentage Gleason pattern 4 or glomeruloid growth pattern for cribriform architecture on radical prostatectomy. The presence of cribriform architecture has been associated with higher percentage Gleason pattern 4 on biopsies. In a cohort of 370 biopsy grade group 2 prostate cancer patients, we found cribriform architecture in 6% of men with <10% Gleason pattern 4, in 22% of men with 10–25% pattern 4 and in 44% of men with 25–50% pattern 4.^7^ Nevertheless, biopsy percentage Gleason pattern 4 was not predictive for cribriform architecture in false‐negative biopsies. This paradoxical outcome could be explained by the low level of concordance between percentage Gleason pattern 4 on biopsy and matched radical prostatectomy specimens in this study. Similarly, glomeruloid Gleason pattern 4, which has been hypothesised to represent a precursor lesion of cribriform growth, was not associated with cribriform architecture in false‐negative biopsies.^16^

Among patients with cribriform architecture, those with large expansive cribriform fields have the worst outcome.^4^ The false‐negative rate of 27% for the large cribriform pattern is significantly less than the rate of 52% for overall cribriform morphology. As 44% of true‐positive biopsies had large cribriform fields on radical prostatectomy compared to only 15% of false‐negative biopsies, this might explain the significantly better biochemical recurrence‐free survival of false‐negative biopsies compared to true‐positive biopsies, in addition to other clinicopathological confounding factors.

The strong points of this study are the detailed histological review of matched biopsies and radical prostatectomies. The study is, however, limited by its low number of patients, the heterogeneity of the study population, including both patients with first‐time diagnosis and progression during active surveillance, and variability of diagnostic work‐up encompassing systematic and/or targeted biopsies, as well as multiparametric MRI assessment. Finally, follow‐up is relatively short, with a median of 27 months.

In conclusion, we demonstrate that 40% of men with biopsy grade group 2 prostate cancer were false‐negative for invasive cribriform and/or intraductal carcinoma. Age and PSA were independent predictors for cribriform architecture in false‐negative biopsies, while patients with false‐negative biopsies more frequently had PI‐RADS score 5 lesions than men with true‐negative biopsies. Multimodal evaluation of biopsy grade group 2 prostate cancer patients could therefore identify men with true cribriform‐negative biopsies who might become eligible for active surveillance.

## Conflict of Interests

The authors declare no conflicts of interest.

## References

[1] Kweldam CF , Kummerlin IP , Nieboer D *et al* Disease‐specific survival of patients with invasive cribriform and intraductal prostate cancer at diagnostic biopsy. Mod. Pathol. 2016; 29; 630–636. 26939875 10.1038/modpathol.2016.49

[2] Truong M , Frye T , Messing E *et al* Historical and contemporary perspectives on cribriform morphology in prostate cancer. Nat. Rev. Urol. 2018; 15; 475–482. 29713007 10.1038/s41585-018-0013-1

[3] Kir G , Sarbay BC , Gumus E *et al* The association of the cribriform pattern with outcome for prostatic adenocarcinomas. Pathol. Res. Pract. 2014; 210; 640–644. 25042388 10.1016/j.prp.2014.06.002

[4] Hollemans E , Verhoef EI , Bangma CH *et al* Large cribriform growth pattern identifies ISUP grade 2 prostate cancer at high risk for recurrence and metastasis. Mod. Pathol. 2019; 32; 139–146. 30349027 10.1038/s41379-018-0157-9 PMC6300553

[5] Dong F , Yang P , Wang C *et al* Architectural heterogeneity and cribriform pattern predict adverse clinical outcome for Gleason grade 4 prostatic adenocarcinoma. Am. J. Surg. Pathol. 2013; 37; 1855–1861. 24145642 10.1097/PAS.0b013e3182a02169

[6] Trudel D , Downes MR , Sykes J *et al* Prognostic impact of intraductal carcinoma and large cribriform carcinoma architecture after prostatectomy in a contemporary cohort. Eur. J. Cancer 2014; 50; 1610–1616. 24703897 10.1016/j.ejca.2014.03.009

[7] Kweldam CF , Kummerlin IP , Nieboer D *et al* Presence of invasive cribriform or intraductal growth at biopsy outperforms percentage grade 4 in predicting outcome of Gleason score 3+4 = 7 prostate cancer. Mod. Pathol. 2017; 30; 1126–1132. 28530220 10.1038/modpathol.2017.29

[8] Kweldam CF , Wildhagen MF , Steyerberg EW *et al* Cribriform growth is highly predictive for postoperative metastasis and disease‐specific death in Gleason score 7 prostate cancer. Mod. Pathol. 2015; 28; 457–464. 25189638 10.1038/modpathol.2014.116

[9] Kweldam CF , Kummerlin IP , Nieboer D *et al* Prostate cancer outcomes of men with biopsy Gleason score 6 and 7 without cribriform or intraductal carcinoma. Eur. J. Cancer 2016; 66; 26–33. 27522247 10.1016/j.ejca.2016.07.012

[10] Sauter G , Steurer S , Clauditz TS *et al* Clinical utility of quantitative Gleason grading in prostate biopsies and prostatectomy specimens. Eur. Urol. 2016; 69; 592–598. 26542947 10.1016/j.eururo.2015.10.029

[11] Corcoran NM , Hovens CM , Hong MK *et al* Underestimation of Gleason score at prostate biopsy reflects sampling error in lower volume tumours. BJU Int. 2012; 109; 660–664. 21895937 10.1111/j.1464-410X.2011.10543.x

[12] Kim KH , Lim SK , Shin TY *et al* Upgrading of Gleason score and prostate volume: a clinicopathological analysis. BJU Int. 2013; 111; 1310–1316. 23452115 10.1111/j.1464-410X.2013.11799.x

[13] Weinreb JC , Barentsz JO , Choyke PL *et al* PI‐RADS Prostate imaging – reporting and data system: 2015, version 2. Eur. Urol. 2016; 69; 16–40. 26427566 10.1016/j.eururo.2015.08.052 PMC6467207

[14] Epstein JI , Egevad L , The Amin MB *et al* 2014 International Society of Urological Pathology (ISUP) Consensus Conference on Gleason Grading of Prostatic Carcinoma: definition of grading patterns and proposal for a new grading system. Am. J. Surg. Pathol. 2016; 40; 244–252. 26492179 10.1097/PAS.0000000000000530

[15] Buyyounouski MK , Choyke PL , McKenney JK *et al* Prostate cancer – major changes in the American Joint Committee on Cancer eighth edition cancer staging manual. CA Cancer J. Clin. 2017; 67; 245–253. 28222223 10.3322/caac.21391 PMC6375094

[16] Lotan TL , Epstein JI . Gleason grading of prostatic adenocarcinoma with glomeruloid features on needle biopsy. Hum. Pathol. 2009; 40; 471–477. 19128819 10.1016/j.humpath.2008.10.002 PMC3484379

[17] Corcoran NM , Hong MK , Casey RG *et al* Upgrade in Gleason score between prostate biopsies and pathology following radical prostatectomy significantly impacts upon the risk of biochemical recurrence. BJU Int. 2011; 108; E202–E210. 21443656 10.1111/j.1464-410X.2011.10119.x

[18] Epstein JI , Feng Z , Trock BJ *et al* Upgrading and downgrading of prostate cancer from biopsy to radical prostatectomy: incidence and predictive factors using the modified Gleason grading system and factoring in tertiary grades. Eur. Urol. 2012; 61; 1019–1024. 22336380 10.1016/j.eururo.2012.01.050 PMC4659370

[19] Masoomian M , Downes MR , Sweet J *et al* Concordance of biopsy and prostatectomy diagnosis of intraductal and cribriform carcinoma in a prospectively collected data set. Histopathology 2019; 74; 474–482. 30160779 10.1111/his.13747

[20] Amin MB , Lin DW , Gore JL *et al* The critical role of the pathologist in determining eligibility for active surveillance as a management option in patients with prostate cancer: consensus statement with recommendations supported by the College of American Pathologists, International Society of Urological Pathology, Association of Directors of Anatomic and Surgical Pathology, the New Zealand Society of Pathologists, and the Prostate Cancer Foundation. Arch. Pathol. Lab. Med. 2014; 138; 1387–1405. 25092589 10.5858/arpa.2014-0219-SA

[21] Montironi R , Hammond EH , Lin DW *et al* Consensus statement with recommendations on active surveillance inclusion criteria and definition of progression in men with localized prostate cancer: the critical role of the pathologist. Virchows Arch. 2014; 465; 623–628. 25316188 10.1007/s00428-014-1668-5

[22] Truong M , Feng C , Hollenberg G *et al* A comprehensive analysis of cribriform morphology on magnetic resonance imaging/ultrasound fusion biopsy correlated with radical prostatectomy specimens. J. Urol. 2018; 199; 106–113. 28728994 10.1016/j.juro.2017.07.037

[23] Prendeville S , Gertner M , Maganti M *et al* Role of magnetic resonance imaging targeted biopsy in detection of prostate cancer harboring adverse pathological features of intraductal carcinoma and invasive cribriform carcinoma. J. Urol. 2018; 200; 104–113. 29408568 10.1016/j.juro.2018.01.081

